# Efficacy and safety of a low-flow veno-venous carbon dioxide removal device: results of an experimental study in adult sheep

**DOI:** 10.1186/cc5082

**Published:** 2006-10-28

**Authors:** Sergio Livigni, Mariella Maio, Enrica Ferretti, Annalisa Longobardo, Raffaele Potenza, Luca Rivalta, Paola Selvaggi, Marco Vergano, Guido Bertolini

**Affiliations:** 1 Department of Anaesthesia and Intensive Care, Ospedale Torino Nord Emergenza San Giovanni Bosco, Piazza del Donatore di Sangue 3, 10154 Turin, Italy; 2 GiViTI Coordinating Center, Laboratory of Clinical Epidemiology, 'Mario Negri' Institute for Pharmacological Research, Villa Camozzi, Via Camozzi 2, 24020 Ranica (Bergamo), Italy

## Abstract

**Introduction:**

Extracorporeal lung assist, an extreme resource in patients with acute respiratory failure (ARF), is expanding its indications since knowledge about ventilator-induced lung injury has increased and protective ventilation has become the standard in ARF.

**Methods:**

A prospective study on seven adult sheep was conducted to quantify carbon dioxide (CO_2_) removal and evaluate the safety of an extracorporeal membrane gas exchanger placed in a veno-venous pump-driven bypass. Animals were anaesthetised, intubated, ventilated in order to reach hypercapnia, and then connected to the CO_2 _removal device. Five animals were treated for three hours, one for nine hours, and one for 12 hours. At the end of the experiment, general anaesthesia was discontinued and animals were extubated. All of them survived.

**Results:**

No significant haemodynamic variations occurred during the experiment. Maintaining an extracorporeal blood flow of 300 ml/minute (4.5% to 5.3% of the mean cardiac output), a constant removal of arterial CO_2_, with an average reduction of 17% to 22%, was observed. Arterial partial pressure of carbon dioxide (PaCO_2_) returned to baseline after treatment discontinuation. No adverse events were observed.

**Conclusion:**

We obtained a significant reduction of PaCO_2 _using low blood flow rates, if compared with other techniques. Percutaneous venous access, simplicity of circuit, minimal anticoagulation requirements, blood flow rate, and haemodynamic impact of this device are more similar to renal replacement therapy than to common extracorporeal respiratory assistance, making it feasible not only in just a few dedicated centres but in a large number of intensive care units as well.

## Introduction

Mechanical ventilation is an essential part of the care provided to the critically ill patients with acute respiratory failure (ARF). Despite the life-saving potential of this assistance, it has disadvantages and complications as well. It has been demonstrated that over-distension and cyclic inflation and deflation of alveoli can damage the alveolar–capillary barrier and initiate or amplify a local and systemic inflammation, so the concept of ventilator-induced lung injury (VILI) was introduced [1].

To prevent VILI, mechanical ventilation was rethought and a lung-protective strategy using lower pressures (plateau pressure <30 cmH_2_O) and smaller tidal volumes (6 to 8 ml/kg of ideal body weight) is now accepted as the standard treatment in patients with ARF [2,3]. Such an approach, however, may result in hypercapnia and acidosis (even within the context of 'permissive hypercapnia,' a pH value lower than 7.2 is not acceptable) [4]. In these cases, the possibility of partially removing carbon dioxide (CO_2_) by using extra-pulmonary devices would be helpful in assisting the lung to maintain acceptable gas exchange.

During the last 3 years, arterovenous pumpless devices, first introduced in 1983, have become the most popular approach for extracorporeal CO_2 _removal (ECCO_2_R) [5]. This relatively simple technique, which uses the arterovenous pressure gradient to force blood through a very low-resistance heparinated circuit, has several advantages: lower anticoagulation requirements, small priming volume, little mechanical damage to blood components, and absence of recirculation. On the other hand, it does not offer direct blood flow control, it increases left-to-right shunt, and it could lead to lower-limb ischaemia due to prolonged arterial cannulation. Moreover, arterial access is not ideal for performing CO_2 _removal within a 'multiorgan support' context, given that both renal replacement and sepsis therapies use veno-venous circuits. The aim of this study was to quantify CO_2 _removal using an extracorporeal membrane gas exchanger placed in a veno-venous pump-driven bypass, collecting preliminary data in an animal model about the efficacy of the system, haemodynamic stability, and occurrence of adverse events.

## Materials and methods

**Figure 1:**
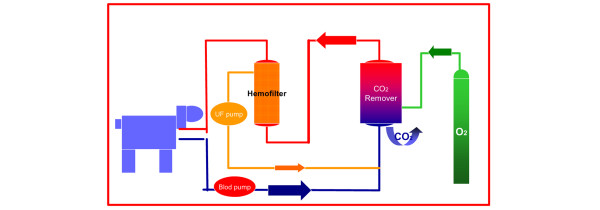
Blood flow through the extracorporeal carbon dioxide (CO_2_) removal circuit. UF, ultra-filtration.

Seven healthy adult female sheep with a mean body weight of 34 kg (range 25 to 41 kg) were used in this study. Animal care and treatment were conducted in accordance with institutional guidelines in compliance with national (Decreto Legislativo n.116, Gazzetta Ufficiale suppl 40, 18 febbraio 1992, Circolare n.8, Gazzetta Ufficiale, 14 luglio 1994) and international (EEC Council Directive 86/609, OJL358-1, December 1987; *Guide for the Care and Use of Laboratory Animals*, U.S. National Research Council, 1996) laws and policies. The protocol was approved by the Ethical Committee of the University of Turin, Italy. Sheep were transported to the laboratory at least two days before the experiment. Anaesthesia was induced (thiopentone 10 to 15 ml/kg) and maintained (isoflurorane 0.8% to 2% and remifentanyl 0.05 to 0.2 μg/kg per minute) during controlled mechanical ventilation (Drägerwerk AG, Lübeck, Germany) after endotracheal intubation, via an intravenous peripheral line. One gram of cephazoline was administered as infection prophylaxis. A femoral artery was cannulated for continuous monitoring of arterial pressure (Datex-Ohmeda, S/5; Datex-Ohmeda, Inc., Madison, WI, USA) and periodic blood sampling for gas analysis (IRMA^®^; Cremascoli & Iris, Milan, Italy). Both jugular veins were cannulated using two 7.5-French catheters for connection with extracorporeal circuit (double lumen cannula did not allow an adequate flow with the sheep in ventral position) and a Swan Ganz catheter (7 French, 4 lumen, 110 cm; Arrow International, Inc., Reading, PA, USA) for periodic monitoring of cardiac output (employing thermodilution technique). Oesophageal temperature was monitored and normothermia (38°C ± 0.5°C) was maintained throughout the experiment. Saline, gelatine, and Ringer's lactate were provided for fluid replacement; low infusion rates of dopamine (2 to 5 μg/kg per minute) and norepinephrine (0.05 to 0.1 μg/kg per minute) were administered when needed as vasopressor support. Gastric tube and vescical catheter were introduced. After the completion of all invasive procedures, ventral position was maintained until the end of the experiment to avoid pulmonary atelectasis and facilitate extubation. Upon achievement of haemodynamic stability during deep anaesthesia, protective ventilation was started with the reduction of minute volume, titrated to reach an arterial partial pressure of carbon dioxide (PaCO_2_) greater than 70 mmHg. After a period of at least 30 minutes without significant variations in PaCO_2_, animals were connected to the CO_2 _removal circuit (Decapsmart, Medica srl, Medolla (Modena), Italy) and treatment was started. No changes in ventilatory setting were made during the treatment. A bolus of 2,000 UI of heparin was administered intravenously, followed by an infusion titrated to maintain ACT (activated clotting time) value between 180 and 220 seconds. Blood was driven through the circuit by a roller non-occlusive pump (Figure 1). Blood flow through the circuit was 300 ml/hour, and warmed gas flow through the oxygenator (0.33 m^2^) (Polystan SAFE Micro Neonatal Oxygenator, Maquet, Rastatt, Germany^®^) was kept constant at 8 l/minute of 100% oxygen. CO_2 _removal treatment was maintained for three hours in five animals. Two sheep were planned to receive longer treatment (12 hours) to assess whether the CO_2 _removal was maintained even after the very first time interval. After the completion of data collection, general anaesthesia was discontinued and the sheep were assisted until complete recovery and extubation.

### Data collection and statistical analysis

Blood samples were taken at the following scheduled times: baseline (that is, immediately before starting treatment [t_0_]); 60 (t_1_), 90 (t_2_), and 210 (t_3_) minutes after t_0_; and 60 minutes after treatment discontinuation (t_4_). Table 1 presents the measurements obtained at each sampling time.

Mean and standard deviation (SD) were used as descriptive statistics for continuous variables. Difference in PaCO_2 _with respect to the baseline was expressed both in absolute and in relative terms. PaCO_2_, cardiac output, and temperature were analysed through repeated measures analysis of variance in the five sheep treated for 4.5 hours (210 + 60 minutes). The contrast matrix was used to assess which of the sampling time values differed significantly from the baseline. Normality and homoschedasticity of the dependent variable distribution were assessed by the normal probability plot and the Spearman correlation coefficient between predicted and absolute values of residuals. A sensitivity analysis of PaCO_2 _was performed on the whole sample of seven sheep, considering only t_0 _to t_3 _sampling times. For each of the two long-treated sheep, mean and 95% confidence interval (CI) of the PaCO_2 _level during treatment were computed. Data were analysed with the SAS^® ^System 9.1.3, SAS Institute Inc., Cary, NC, USA.

## Results

**Figure 2:**
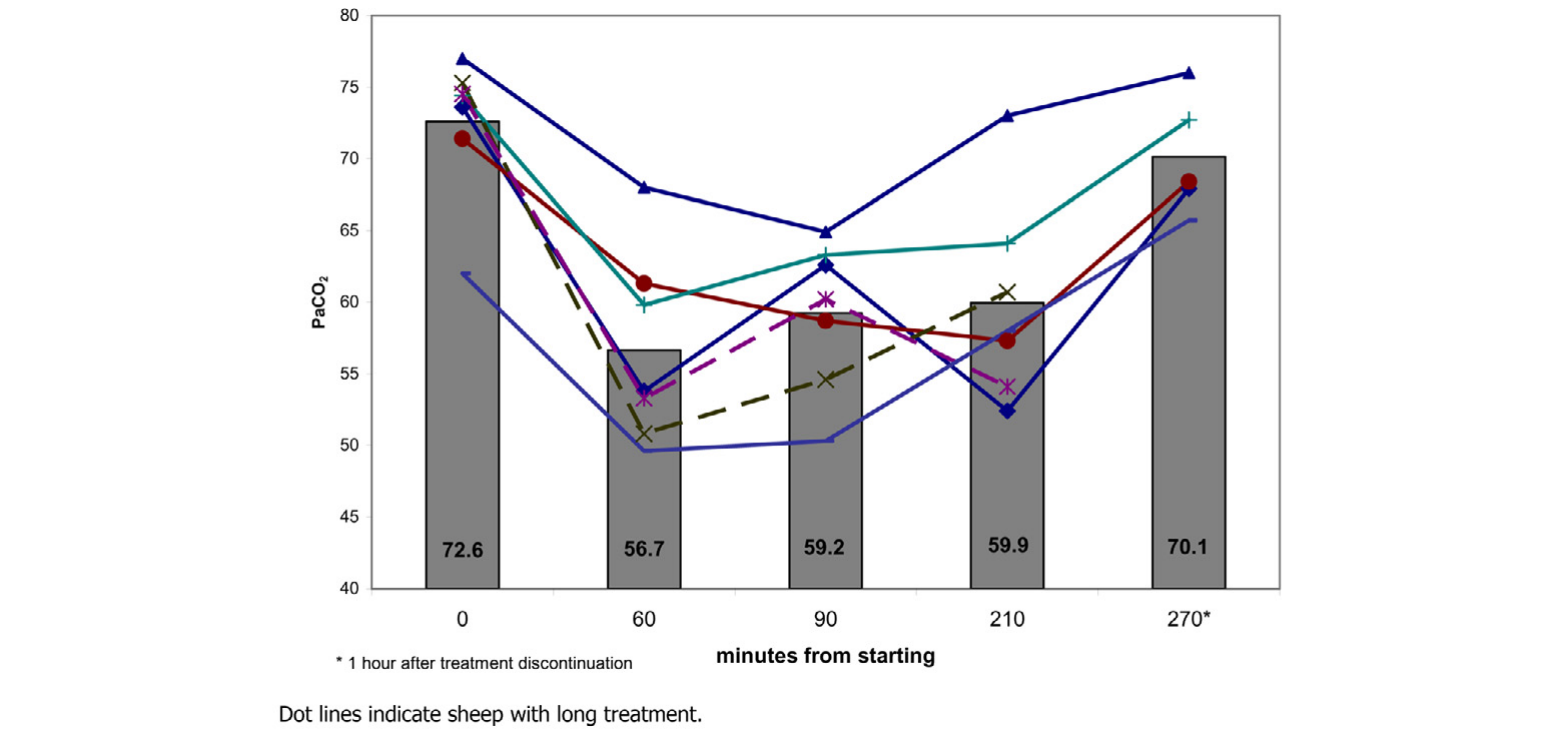
Mean arterial partial pressure of carbon dioxide (PaCO_2_) levels at baseline, 60, 90, and 210 minutes from starting, and 60 minutes from discontinuation. Dotted lines indicate sheep with long treatment.

Considering all seven sheep, we observed an average (SD) relative reduction in PaCO_2_, with respect to the baseline, of 21.9% (7.7%) at 60 minutes, 18.4% (4.4%) at 90 minutes, and 17.3% (9.3%) at 210 minutes from starting treatment. After treatment discontinuation and without any variation in ventilatory setting, PaCO_2 _returned to its baseline level (Figure 2).

As expected with a low-flow bypass, no significant effects occurred in oxygenation. Mean cardiac output and body temperature did not significantly change from t_0 _to t_4_, nor did extracorporeal blood flow (EBF), which was actually kept constant during the treatment. Thus, the EBF-to-cardiac output ratio was persistently approximately 5% (4.5% to 5.3%). All other parameters collected remained constant during the treatment.

The repeated measures analysis of variance of the five sheep receiving a short treatment course clearly indicates that PaCO_2 _was significantly and persistently removed by the treatment and that suddenly after the treatment discontinuation it returned to its pre-treatment level (Table 2). The other parameters tested with this analysis (cardiac output and temperature) were not influenced by the treatment. The sensitivity analysis on PaCO_2 _considering the first three sampling times of all seven sheep strengthened this result, given that the differences with the baseline (Figure 2) were all highly significant (*p *= 0.0004 at 60 minutes, *p *< 0.0001 at 90 minutes, and *p *= 0.003 at 210 minutes).

**Figure 3:**
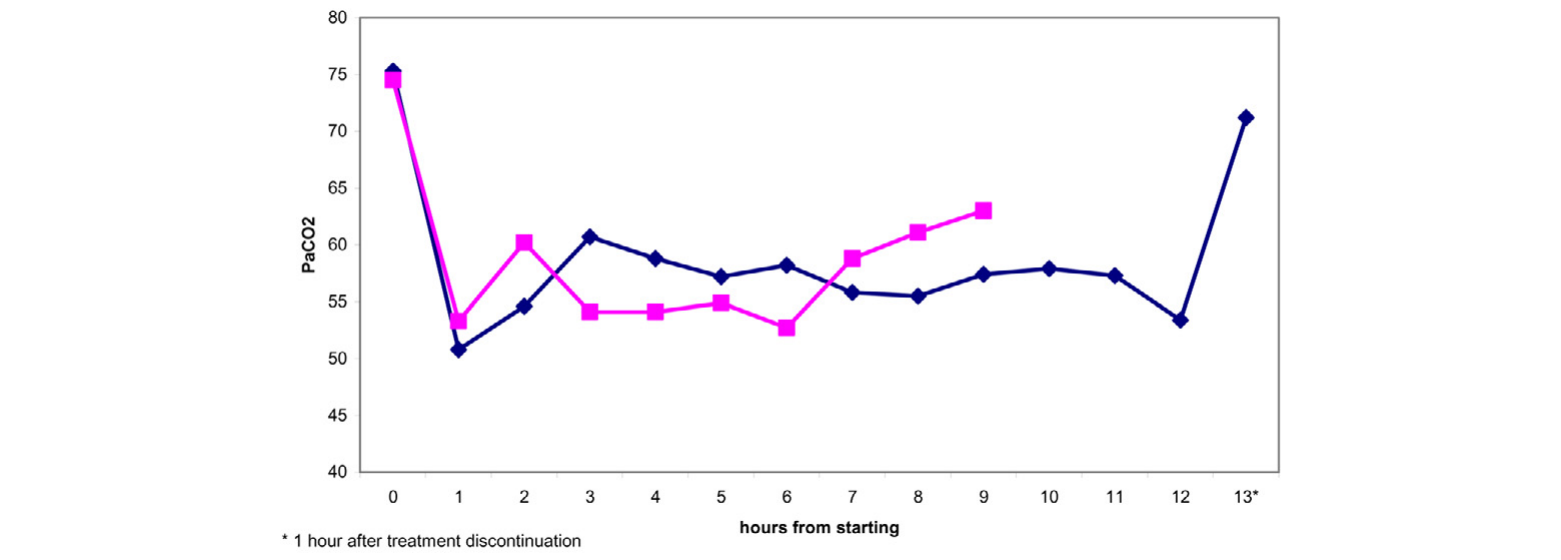
Arterial partial pressure of carbon dioxide (PaCO_2_) levels during two longer treatments.

Two sheep were maintained under treatment for a longer time to appreciate the persistency of CO_2 _removal. Although in these two cases we planned to continue the treatment for 12 hours, in one case we were forced to stop the experiment after 9 hours due to an electricity blackout. In this case, we missed the post-treatment sampling. Figure 3 shows the PaCO_2 _levels in these two cases. The average PaCO_2 _values during the treatment course for the two sheep were 56.5 mmHg (95% CI = 55.0 to 58.0) and 56.9 mmHg (95% CI = 54.4 to 59.4), whereas their respective baseline levels were 75.3 and 74.5 mmHg.

No adverse events in terms of bleeding, clotting of circuit, severe haemodynamic instability, or venous embolism were observed. All animals involved in the study survived and left the laboratory in good health within one week.

## Discussion

Although many pharmacologic and nonpharmacologic interventions have been developed to assist lung function during acute lung injury, none of them demonstrated a clear superiority and became the standard. Pharmacologic approaches included nitric oxide inhalation, surfactant replacement therapy, antioxidants, prostaglandins, and corticosteroids. Non-pharmacologic interventions are essentially represented by prone positioning, protective ventilation, PEEP (positive end-expiratory pressure), fluid management, and extracorporeal techniques, from extracorporeal membrane oxygenation (ECMO) to ECCO_2_R. Unfortunately, no large randomised trial on the efficacy of extracorporeal lung assist is available, though different case series showed encouraging results in terms of survival rates among high-risk patients [6,7].

ECMO is the first procedure proposed, but it has the disadvantages of an increased bleeding risk (even if reduced after the introduction of percutaneous cannulation techniques and heparin-coated circuits) and the requirement of specialised perfusionist staff, along with an experienced multidisciplinary team. Indeed, ECMO is a complex and invasive procedure that can be safely run in just a few dedicated centres with extensive research experience. At the time of the first studies in the 1970s, the idea of 'lung rest' (that is, using low tidal volume) [8] had no scientific rationale, and the modern concepts of 'baby lung' (that is, lower dimensions of the normally aerated tissue) [9], VILI, and protective ventilation did not yet exist. More recently, the original target of maintaining normal blood gas values has become performing the most possible gentle ventilation [9]. This could be done with the introduction of ECCO_2_R, dissociating oxygenation (via the native lungs) from CO_2 _removal (using veno-venous extracorporeal bypass) [10-12]. Later on, the concept of 'permissive hypercapnia' consistently diminished the requirement of CO_2 _removal as an indication for extracorporeal lung support [4].

Today, the new concept of 'permissive hypoxemia' has emerged [13]. In this context, hypercapnia and acidosis are no longer seen as harmful but even useful in improving tissue oxygenation by right-shifting the oxyhaemoglobin dissociation curve. Nonetheless, when hypercapnia and moderate hypoxiemia are tolerated as part of the clinical strategy, a method to reliably reduce PaCO_2 _would still be very helpful in performing the most feasible lung-protective strategy.

A variety of recent studies have investigated the efficacy and safety of pumpless arterovenous devices to remove CO_2 _[14-17], which offer several advantages over ECMO: reduced bleeding risk, less time consumption, lower cost, no mechanical damage of blood components, and no need for a perfusionist staff. But these techniques also have their disadvantages, the most common being ischaemia of the lower limb after prolonged femoral arterial cannulation, increase of left-to-right shunt (thus excluding patients with cardiac failure), and no direct blood flow control.

Our study was designed to evaluate the efficacy and safety of a veno-venous CO_2 _removal device in an animal model. By using an EBF of up to 5% of cardiac output, we succeeded in reducing PaCO_2 _by 17% to 22% without variations in ventilatory setting. We observed a respiratory alkalosis in the bypassed blood (mean PaCO_2 _post-filter, 22.5 mmHg; pH post-filter, 7.67; HCO_3_^- ^post-filter, 26.0 mmol/l). However, due to the low flow of the bypass compared with the cardiac output, this did not translate into a systemic alkalosis (overall mean PaCO_2_, 59.8 mmHg; pH, 7.31; HCO_3_^-^, 29.4 mmol/l). The CO_2 _reduction was consistent at every scheduled time during the experiment, was maintained even in longer treatments (9 and 12 hours), and was not influenced by CO_2 _production, as deep anaesthesia was maintained and no significant variations in cardiac output and body temperature were observed.

No adverse events in terms of bleeding, clotting of circuit, haemodynamic instability, or venous embolism were observed, thus showing the apparent safety of this technique in animal models. However, apart from the small sample size, one of the major limits of our study in this regard is represented by the shortness of treatments. Given that this technique in the clinical setting could be maintained for several days, the occurrence of long-term adverse events could consequently be different.

## Conclusion

The results we obtained are very promising, and the possibility of applying this technique to real patients is nearer. This should be viewed as an important achievement because, regardless of whether the first applications confirmed our results, this procedure could become the first choice for ECCO_2_R in patients with ARF, particularly in intensive care units experienced in depurative techniques. Increasingly, extracorporeal techniques have become a successful option for supporting different organs, from renal and liver functions to acid-base and fluid-balance control [18], to sepsis treatment [19]. In this context, different approaches, such as continuous veno-venous haemofiltration, coupled plasmafiltration adsorption, and CO_2 _removal, can be performed simultaneously in what is called 'multiorgan support therapy' [20].

The very first step of this process can be considered completed. According to the model of pharmacological research, clinical studies looking at toxicity (phase I) and biological activity (phase II) should precede a large-scale randomised controlled trial before the technique can be introduced in the real world, but the effort seems worth it.

## Key messages

• Extracorporeal lung assist may play a key role in preventing VILI.

• Low-flow veno-venous bypass may obtain a significative reduction of PaCO_2_.

• The simplicity of this technique makes the device more similar to renal replacement therapy than to common extracorporeal respiratory assistance.

## Abbreviations

ARF = acute respiratory failure; CI = confidence interval; CO_2 _= carbon dioxide; EBF = extracorporeal blood flow; ECCO_2_R = extracorporeal carbon dioxide removal; ECMO = extracorporeal membrane oxygenation; PaCO_2 _= arterial partial pressure of carbon dioxide; SD = standard deviation; VILI = ventilator-induced lung injury.

## Competing interests

The authors declare that they have no competing interests.

## Authors' contributions

SL performed study conception and design, experiments, interpretation of data, and manuscript drafting. MM performed study design, experiments, and data acquisition and analysis. EF, AL, RP, LR, and PS conducted experiments and data acquisition. MV conducted experiments, interpretation of data, and manuscript drafting. GB performed study conception and design, statistical analysis and interpretation of data, and revision of manuscript. All authors read and approved the final manuscript.

## Figures and Tables

**Table 1:** Measurements taken at each sampling time

Arterial blood gases	PaO_2_, PaCO_2_, SaO_2_
Venous blood gases	PvO_2_, PvCO_2_, SvO_2_
Pre- and post-filter blood	CO_2_
End tidal	CO_2_
Oxygenator parameters	Oxygen flow through oxygenator
	Circuit blood flow
	Circuit blood pressure before and after oxygenator
	Sweep gas PCO_2_
Coagulation parameters	ACT, heparin infusion as units per hour
Haemodynamic parameters	Systolic and diastolic arterial pressure
	Heart rate
	Cardiac output
	Central venous pressure
	Pulmonary artery wedge pressure
	Pulmonary artery pressure
	Systemic and pulmonary vascular resistance
Clinical parameters	Diuresis
	Body temperature
	Fluid balance
Ventilator setting	FiO_2_
	Tidal volume
	Respiratory rate
	PEEP
	Plateau pressure
	Peak pressure

ACT, activated coagulation time; CO_2_, carbon dioxide; FiO_2_, fraction of inspired oxygen; PaCO_2_, (arterial) partial pressure of carbon dioxide; PaO_2_, (arterial) partial pressure of oxygen; PCO_2_, partial pressure of carbon dioxide in the sweep gas; PEEP, positive end-expiratory pressure; PvCO_2_, mixed venous carbon dioxide pressure; PvO_2_, mixed venous oxygen pressure; SaO_2_, oxyhaemoglobin saturation; SvO_2_, mixed venous oxygen saturation.

**Table 2:** Parameters at baseline and during the course of treatment for five sheep receiving short-term treatment

Parameter	Baseline, mean (SD)	Time point from baseline							
		
		1		2		3		4 (1 hour after treatment discontinuation)	
		
		Mean (SD)	Contrast with the baseline	Mean (SD)	Contrast with the baseline	Mean (SD)	Contrast with the baseline	Mean (SD)	Contrast with the baseline
PaCO_2 _(mmHg)	71.7 (5.8)	58.5 (7.1)	*p *= 0.002	60.0 (5.9)	*p *< 0.0001	61.0 (7.9)	*p *= 0.03	70.1 (4.1)	*p *= 0.37
Cardiac output (l/min)	6.6 (0.9)	5.8 (1.2)	*p *= 0.32	5.7 (1.1)	*p *= 0.21	6.4 (1.7)	*p *= 0.82	5.8 (1.3)	*p *= 0.27
Temperature (°C)	38.5 (0.5)	38.1 (1.0)	*p *= 0.15	38.3 (1.2)	*p *= 0.68	37.8 (1.7)	*p *= 0.30	37.8 (1.5)	*p *= 0.27

PaCO_2_, arterial partial pressure of carbon dioxide; SD, standard deviation.
